# Does MRI help in the pre - operative evaluation of pelvic fracture urethral distraction defect? - a pilot study

**DOI:** 10.1590/S1677-5538.IBJU.2016.0252

**Published:** 2017

**Authors:** Rajadoss Muthukrishna Pandian, Nirmal Thampi John, Anu Eapen, B. Antonisamy, Antony Devasia, Nitin Kekre

**Affiliations:** 1 Department of Urology, Christian Medical College and Hospital, Vellore, Tamil Nadu, India;; 2 Department of Radiology, Christian Medical College and Hospital, Vellore, Tamil Nadu, India;; 3 Department of Biostatistics, Christian Medical College and Hospital, Tamil Nadu, India

**Keywords:** Surveys and Questionnaires, Pelvis, Magnetic Resonance Imaging, Erectile Dysfunction

## Abstract

**Objectives:**

To study the usefulness of MRI in preoperative evaluation of PFUDD. Can MRI provide additional information on urethral distraction defect (UDD) and cause of erectile dysfunction (ED)?

**Materials and Methods:**

In this prospective study, consecutive male patients presenting with PFUDD were included from Feb 2011 till Dec 2012. Those with traumatic spinal cord injury and pre-existing ED were excluded. Patients were assessed using IIEF questionnaire, retrograde urethrogram and micturating cystourethrogram (RGU+MCU) and MRI pelvis. Primary end point was erectile function and secondary end point was surgical outcome.

**Results:**

Twenty patients were included in this study. Fourteen patients (70%) were ≤40years; fifteen patients (75%) had ED, seven patients (35%) had severe ED. MRI findings associated with ED were longer median UDD (23mm vs. 15mm, p=0.07), cavernosal injury (100%, p=0.53), rectal injury (100%, p=0.53), retropubic scarring (60%, p=0.62) and prostatic displacement (60%, p=0.99). Twelve patients (60%) had a good surgical outcome, five (25%) had an acceptable outcome, three (15%) had a poor outcome. Poor surgical outcome was associated with rectal injury (66.7%, p=0.08), cavernosal injury (25%, p=0.19), retropubic scarring (18.1%, p=0.99) and prostatic displacement (16.7%, p=0.99). Five patients with normal erections had good surgical outcome. Three patients with ED had poor outcome (20%, p=0.20).

**Conclusions:**

MRI did not offer significant advantage over MCU in the subgroup of men with normal erections. Cavernosal injury noted on MRI strongly correlated with ED. Role of MRI may be limited to the subgroup with ED or an inconclusive MCU.

## INTRODUCTION

Posterior urethral injury complicates up to 25% of pelvic fractures arising from blunt pelvic trauma (1). Since majority of patients with traumatic urethral injuries are younger than 40 years, ED is a devastating complication encountered in up to 54% of these individuals (2, 3). Patients with PFUDD (pelvic fracture urethral distraction defect) are routinely evaluated with combined RGU (retrograde urethrogram) and MCU (micturating cystourethrogram). Their limitations include the 2 dimensional images and the non-visualization of prostatic urethra in some patients. MRI pelvis can be helpful in studying the distorted pelvic anatomy and planning surgical approach as well as to help evaluation of erectile dysfunction (4-6). It has been suggested that certain MRI findings have a higher association with ED (7). MR urethrogram has been suggested to show structural details of urethra as well as periurethral tissues with 3-dimensional orientation (8).

The purpose of this study was to find out whether MRI imaging would offer any additional information helpful in the pre-operative planning, counseling and management of PFUDD, especially in the subgroup of men with ED.

## MATERIAL AND METHODS

### Study design

A prospective study was carried out between February 2011 and December 2012. Following Institutional Review Board and Ethics Committee approval, consecutive men presenting with pelvic fracture urethral distraction defect (PFUDD) scheduled for primary urethral reconstruction were recruited in this study. Patient with traumatic spinal cord injury, pre-existing ED, previous operative interventions for PFUDD, co-morbid conditions like diabetes and hypertension with end organ damage were excluded.

### Pre-operative evaluation

Erectile function was assessed using a validated questionnaire (International Index of Erectile Function-IIEF); MRI pelvis was performed prior to urethral reconstruction. Patients were classified according to the Erectile Function domain of International Index of Erectile function (IIEF-EF) into three groups: normal erectile function (≥25), mild to moderate ED [7-24] and severe ED (≤6). The final comparison was done between those with normal erectile function and those with ED (moderate and severe ED).

### MRI pelvis

**Figure 1:**
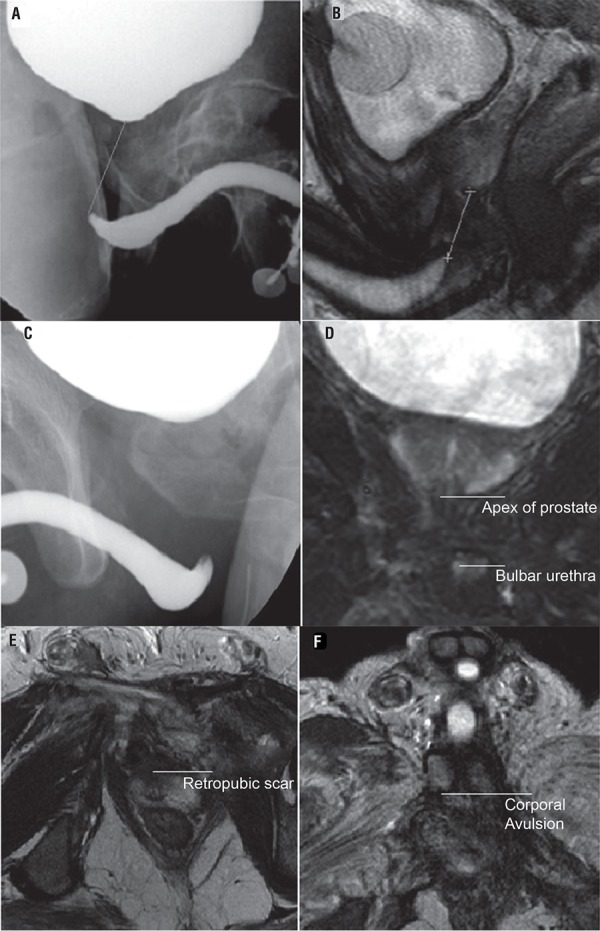
The figure shows urethral defect on MCU (A) and MRI (B); prostatic displacement on MCU (C) and MRI (D); retropubic scarring on MRI (E); and corporal avulsion on MRI (F).

MRI pelvis was done using Philips intera achieva 3.0 tesla. Anterior urethra was distended with normal saline using a 12Fr. Foley catheter placed under aseptic precautions with a partially inflated bulb (0.5-1mL) placed at the fossa navicularis. 2% Xylocaine jelly was used for local anaesthesia. Suprapubic catheter was clamped 30 minutes prior to the study to allow natural distension of the bladder. The image series obtained included: T2WI sagittal, axial, coronal; STIR_Long TE/RA, SshTSE, SPAIR, SENSE. TR: 3500ms, TE 90.0ms, ST 3.0mm. The following parameters were assessed by the same radiologist: length of urethral defect (Figures 1A and B) (distance between prostatic apex and the most proximal portion of the bulbar urethra), direction of prostatic displacement (Figures 1C and D) (superior, posterior, or lateral), and extent of scar tissue (Figure-1E) (retropubic, prostatic, peri-prostatic, or subprostatic). Presence of bladder base fistula, rectal injury or cavernosal injury (Figure-1F) was documented.

### Surgical outcome

The operative outcome was categorized into 3 groups based on the previously published data from this department (9). The 3 groups were: good outcome with Qmax >15mL/sec, acceptable outcome with Qmax >15mL/sec after a single endoscopic internal urethrotomy and failure when Qmax <15mL/sec. Further comparisons were made between MRI findings, erectile function and surgical outcome.

#### Statistical analysis

Statistical analysis was performed using SPSS version 16 (IBM Corporation, USA). All categorical variables were summarized as counts and percentages and continuous variables as mean and standard deviation or Median and Range. Fisher’s exact test was used for testing the association between categorical variables and Wilcoxon rank sum test was used for comparing non-normally distributed continuous variable by groups.

## RESULTS

Twenty patients with traumatic posterior urethral injury were recruited during the study period. The median age at presentation was 34 years, (range of 17-61 years) (Table-1). Road traffic accident was the mode of injury in 18 (90%) patients. All patients underwent trocar suprapubic catheterization with 14Fr Foley’s catheter during the emergency admission. None had undergone an attempt of primary realignment. Three who had associated anorectal injuries underwent diversion colostomy. Pelvic fracture type A was the commonest (11/20). One patient underwent external fixation of pelvic fracture. Urethral reconstruction was performed after 3 months. Evaluation using IIEF questionnaire showed ED in 15 patients (75%), while 7 patients (35%) had severe ED.

**Table 1:** Baseline characteristics of patients with pelvic fracture urethral distraction defect (n=20).

Baseline characteristic	Value	Number (%)
Age at presentation (years)	Median (range)	34 (17-61)
	<40 years (%)	14 (70.0)
Mode of injury	Road traffic accidents	18 (90.0)
	Fall from train	1 (5.0)
	Crushed by collapsing wall	1 (5.0)
Type of pelvic fracture	Tile A	11 (55.0)
	Tile B	2 (10.0)
	Tile C	7 (35.0)
IIEF score (EF domain)	Normal (25-35)	5 (25.0)
	Moderate erectile dysfunction (7-24)	8 (40.0)
	Severe erectile dysfunction (≤6)	7 (35.0)

### Urethral distraction defect (UDD)

The posterior urethra was not visualized in 4 men; in this study all 4 patients had ED. The median UUD on MCU in those with ED was longer than those with normal erectile function (40mm vs. 13mm, p=0.039). The median UUD on MRI in those with ED was longer than those with normal erectile function (23mm vs. 15mm, p=0.07). The median intra-op UDD correlated with median MRI UDD more than the median MCU UDD; especially in those with erectile dysfunction (20mm vs. 23mm vs. 40mm).

### MRI findings

Prostatic displacement was present in 12 patients (60%). Retropubic scarring was seen in 11 patients (55%). Injury to corpora cavernosa was seen in 4 patients (20%) (Figure-1). Three patients (15%) had recto-urethral fistula. About 90% of patients with ED had either retropubic scarring or prostatic displacement. MRI findings associated with ED were cavernosal injury (100%, p=0.53), rectal injury (100%, p=0.53), retropubic scarring (60%, p=0.62) and prostatic displacement (60%, p=0.99), though this did not reach statistical significance. ED was seen in all patients with either cavernosal injury or rectal injury. The MRI findings did not change the surgical management.

### Surgical outcome

All twenty patients underwent anastomotic urethroplasty by progressive perineal approach. Twelve patients had a good operative outcome. Five patients with poor flow had soft strictures, requiring cystoscopy and dilation once as outpatient. They were advised self calibration. Subsequently, they had a satisfactory urine flow with Qmax >15mL/sec. Three patients failed to void normally following catheter removal. They all underwent suprapubic catheter placement. Two patients underwent a redo anastomotic urethroplasty with good outcome. Third patient was lost to follow-up.

All five patients who reported normal erectile function post trauma had good surgical outcome. Seven (46.7%) out of the fifteen patients with ED had a good outcome, while five patients (33%) had an acceptable outcome and three patients (20%, p=0.20) had a poor outcome. MRI findings were compared with the surgical outcome (Table-2). MRI findings associated with poor surgical outcome were rectal injury (66.7%, p=0.08), cavernosal injury (25%, p=0.19), retropubic scarring (18.1%, p=0.99) and prostatic displacement (16.7%, p=0.99). Two out of three patients with rectal injury had poor surgical outcome, though this did not reach statistical significance.

**Table 2:** Comparison of surgical outcome with erectile function and MRI findings (n=20).

	Surgical Outcome			P value‡

	Good (n=12)	Acceptable (n=5)	Failure (n=3)	
**Erectile function**				
Normal (n=5)	5 (100.0)	0	0	0.20
Moderate and Severe ED (n=8)	7 (46.7)	5 (33.3)	3 (20.0)	
**MRI findings**				
**Retropubic scarring (n=11)**				
Yes	6 (54.6)	3 (27.3)	2 (18.1)	0.99
No	6 (66.7)	2 (22.2)	1 (11.1)	
**Prostatic displacement (n=12)**				
Yes	7 (58.3)	3 (25.0)	2 (16.7)	0.99
No	5(62.5)	2 (25.0)	1 (12.5)	
**Cavernosal Injury (n=4)**				
Yes	1 (25.0)	2 (50.0)	1 (25.0)	0.19
No	11 (68.9)	3 (18.8)	2 (12.5)	
**Rectal injury (n=3)**				
Yes	1 (33.3)	-	2 (66.7)	0.08
No	11 (64.7)	5 (29.4)	1 (5.9)	

^‡^ p value is obtained using fisher’s exact test

## DISCUSSION

Younger age of presentation noted in our study correlated with the review by Kulkarni et al. They found higher proportion of children and adolescents presenting with PFUDD in India when compared to Italy (25.6% vs. 8%) (10). Urethral distraction defects occur mainly in Tile B and C pelvic fractures (11). In our study, Tile A was the commonest. ED was defined by NIH consensus development conference as “the inability to achieve an erect penis as part of overall multifaceted process of male sexual performance, pelvic fracture being a major risk factor” (12, 13). In a cross-sectional study of male sexual function after pelvic ring fractures using the International Index for Erectile Function (IIEF), pubic diastasis was related to impaired erectile function and overall satisfaction (14).

High incidence of ED (75%) in this group was comparable with the study by Shenfeld et al. (15) in which 72% had ED. Anger et al. reported ED of some degree in 54% of patients with PFUDD and severe ED in 30% (3). Corriere et al. reported prevalence of ED following trauma as 25% (50/197) (16). Koraitim reported prevalence of ED after traumatic posterior urethral injury in 44 out of 110 (40%) patients who were sexually potent (14). King reported prevalence of ED in 42% of patients with PFUDD when compared to 5% of patients with pelvic fracture alone (17). Introduction of validated IIEF questionnaire in 1997 has helped in a more objective and detailed assessment of erectile dysfunction (18). Lack of such objective assessment in the past could explain the wide variation in the prevalence of erectile dysfunction prior to this. Evaluation of nocturnal tumescence and rigidity has revealed ED in up to 84% (19). The cause of ED following PFUUD was speculated to be neurovascular injury (15). Mark et al. reviewed 92 patients and found ED in 62%. Urethral reconstruction did not lead to ED in potent man. In those who had ED, self-injection using intracavernous vasoactive drugs was successful in 24 out of 27 patients (89%), which could suggest that the etiology was neurological (20).

In a review of MRI done on 27 patients with PFUDD by Narumi et al., 95% of those with corporal avulsion had ED while 83% had normal erection in the absence of these findings (4). In another study by Koraitim et al., 21 patients with PFUDD were assessed using MRI combined with antegrade urethrography. They found avulsion of cavernosa from the ischium as well as lateral displacement of prostate in all patients with ED (7). Proximity of cavernosal nerves and internal pudendal arteries to the prostatic apex makes this observation interesting (15). In our study, MRI showed prostatic displacement in 12 patients and injury to corpora cavernosa in 4 patients. Erectile dysfunction was present in all patients with cavernosal injury. ED caused by cavernosal injury is unlikely to respond to pharmacological interventions and would require penile prosthesis. This has a significant impact on pre-operative counselling and management of ED.

MRI helps identify the exact urethral distraction defect especially when the posterior urethra is not visualised on micturating cystourethrogram. The degree and direction of prostatic displacement becomes evident. MRI also reveals the presence of concomitant rectal injury. MR urethrogram has been reported to be more reliable than combined RGU and MCU in measuring the length of obliterative urethral strictures (21). This was noted in our study in the subgroup of men with erectile dysfunction and when prostatic urethra was not visualized on MCU. MRI has been suggested to have a significant impact on pre-op decision making, counselling and the appropriate surgical approach (7). This was not seen in our study.

MRI provides detailed three-dimensional images of the urethral distraction defect. In men with normal erections, MRI findings did not have a significant impact on the pre-operative decision making or counselling. In those with erection dysfunction, presence of cavernosal injury noted on the MRI added value to preoperative counselling and management of ED. Disadvantages of MRI pelvis include the higher cost, contraindication in those with ferromagnetic implants and longer duration of study in enclosed space. Considering these factors, there is little advantage of preoperative MRI in the evaluation of PFUDD in men with normal erections when the posterior urethra was visualized on MCU. Presence of ED based on the IIEF questionnaire and non-visualization of posterior urethra on MCU can help us decide on the use of preoperative MRI in PFUDD.

Although our study includes a small group of patients, we believe that this prospective study gives directions for further research.

## CONCLUSIONS

MRI provides detailed three-dimensional images of the urethral distraction defect. MRI did not offer significant advantage over MCU in the pre-operative evaluation of PFUDD in the subgroup of men with normal erection. Cavernosal injury noted on MRI strongly correlated with ED, added value to pre-operative counseling and management of ED. Role of MRI may be limited to the subgroup with ED and those with non-visualized posterior urethra on MCU.

## ARTICLE INFO

Int Braz J Urol. 2017; 43: 127-33
